# The role of perioperative sedative anesthetics in preventing postoperative delirium: a systematic review and network-meta analysis including 6679 patients

**DOI:** 10.1186/s12872-024-03783-5

**Published:** 2024-03-06

**Authors:** Jin-Xiang Huang, Shan-Shan Zhang, Shu-Xian Wang, Da-Shuang Xi, Fang-Ru Luo, Cheng-Jiang Liu, Hong Li

**Affiliations:** 1 Department of Anesthesiology, Second Affiliated Hospital of Army Medical University, Chongqing, China; 2 Department of General Medicine, Affiliated Anqing First People’s Hospital of Anhui Medical University, Anqing, China

**Keywords:** Postoperative delirium, Dexmedetomidine, Sevoflurane, Midazolam, Propofol

## Abstract

**Objective:**

Postoperative delirium is a common and debilitating complication that significantly affects patients and their families. The purpose of this study is to investigate whether there is an effective sedative that can prevent postoperative delirium while also examining the safety of using sedatives during the perioperative period.

**Methods:**

The net-meta analysis was used to compare the incidence of postoperative delirium among four sedatives: sevoflurane, propofol, dexmedetomidine, and midazolam. Interventions were ranked according to their surface under the cumulative ranking curve (SUCRA).

**Results:**

A total of 41 RCT studies involving 6679 patients were analyzed. Dexmedetomidine can effectively reduce the incidence of postoperative delirium than propofol (OR 0.47 95% CI 0.25–0.90), midazolam (OR 0.42 95% CI 0.17-1.00), normal saline (OR 0.42 95% CI 0.33–0.54) and sevoflurane (OR 0.39 95% CI 0.18–0.82). The saline group showed a significantly lower incidence of bradycardia compared to the group receiving dexmedetomidine (OR 0.55 95% CI 0.37–0.80). In cardiac surgery, midazolam (OR 3.34 95%CI 2.04–5.48) and normal saline (OR 2.27 95%CI 1.17–4.39) had a higher rate of postoperative delirium than dexmedetomidine, while in non-cardiac surgery, normal saline (OR 1.98 95%CI 1.44–2.71) was more susceptible to postoperative delirium than dexmedetomidine.

**Conclusion:**

Our analysis suggests that dexmedetomidine is an effective sedative in preventing postoperative delirium whether in cardiac surgery or non-cardiac surgery. The preventive effect of dexmedetomidine on postoperative delirium becomes more apparent with longer surgical and extubation times. However, it should be administered with caution as it was found to be associated with bradycardia.

**Supplementary Information:**

The online version contains supplementary material available at 10.1186/s12872-024-03783-5.

## Introduction

Postoperative delirium, also known as postoperative cognitive dysfunction (POCD), refers to delirium that occurs after surgery and is a common complication with an incidence rate of 70% [1–3]. It typically manifests 1–3 days after surgery and can last up to 2–5 days. While it can be completely relieved in most cases, recent studies suggest a strong association between postoperative delirium and the development of long-term cognitive and non-cognitive diseases [4], including post-traumatic stress disorder [5]. Moreover, postoperative delirium can lead to prolonged hospitalization, increased hospitalization costs, reduced quality of life, and increased mortality and complications [6], which poses a significant burden to both society and families. The incidence of delirium is higher in the elderly population, and with an aging population, postoperative cognitive dysfunction has become an increasingly hot topic [7–9]. Currently, there is no clear treatment for postoperative delirium, and it is generally managed through symptomatic treatment and the use of sedative adjuvants. Although some progress has been made in the diagnosis and treatment of the disease [10–12], there is still much debate surrounding the prevention of postoperative delirium.

Some researchers argue that sedatives, such as midazolam, may be a contributing factor in postoperative delirium [13, 14]. However, the article suggests that dexmedetomidine can help to prevent postoperative delirium [15], although the effects on circulation and heart rate remain a controversial issue. Previous studies have shown that the use of propofol and sevoflurane for preventing postoperative delirium has produced mixed results [16–19]. Given the variety of sedatives available to anesthesiologists, it is crucial to choose the appropriate one that can minimize the risk of postoperative delirium.

A meta-analysis is a powerful tool that can overcome the limitations of sample size in individual studies. Net meta-analysis takes this approach one step further by comparing different interventions through the combination of direct and indirect evidence, ultimately ranking them to identify the most effective treatment measures. The purpose of our study was to evaluate the preventive effects of four commonly used anesthetic drugs, including sevoflurane, propofol, dexmedetomidine, and midazolam, on postoperative delirium. By synthesizing the available evidence, we aimed to provide a comprehensive and robust evaluation of the relative efficacy and safety of these anesthetic drugs in preventing postoperative delirium.

## Methods

### Eligibility standards

To ensure the rigor and reliability of our analysis, we followed the guidelines and recommendations of PIRIMA (Preferred Reporting Items for systematic reviews and meta-Analyses). The registration ID of PROSPERO is CRD42023426641.

The studies were deemed eligible for inclusion in this review only if they met the specific criteria established for this study:

- population: we restricted our study to adult patients aged 18 years or older and excluded studies involving individuals with pre-existing brain diseases, dementia, delirium, cerebral infarction, or cerebral ischemia.
- intervention: comparison between dexmedetomidine, sevoflurane, midazolam, and propofol, saline group. the experimental group treated with placebo or one of the the three drugs can be used as a control group. Other drugs are not included.
- outcome: The primary outcome measure of our review was the incidence of postoperative delirium. To control for potential confounding factors, we limited our analysis to cases where delirium was diagnosed within seven days of surgery. Only studies that utilized reliable tools for diagnosing delirium, such as the Confusion Assessment Method (CAM), Mini-Mental State Examination (MMSE), Nursing Delirium Screening Scale (Nu-DESC scoring), Delirium Rating Scale (DRS), and Memorial Delirium Assessment Scale were included in our review.
- study design: studies were RCT studies.

### Search strategy

We conducted a comprehensive search on multiple databases, including PubMed, EMBASE, and Cochrane Central, for relevant literature published before the end of december 2022. Using the search terms: the Subject words and free words of postoperative delirium AND dexmedetomidine OR propofol OR sevoflurane OR midazolam. During the full-text screening process, non-random experiments are typically excluded as they may have a higher risk of selection bias and thus may affect the reliability and generalizability of study outcomes. By adopting a rigorous and systematic approach to data collection, we aimed to reduce the risk of selection bias and enhance the validity of our findings.

### Study selection

**Fig. 1:**
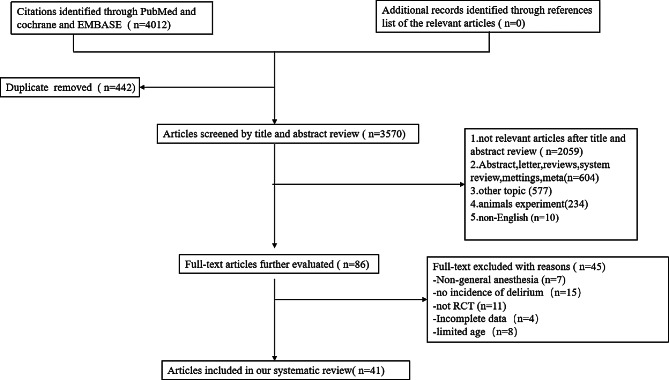
The flowchart of the searching strategy

To manage the literature search process, we utilized EndNote X9 software to import and automatically remove any duplicate documents. We then organized and screened the articles based on our predetermined criteria. Case reports, letters, and records of meetings were excluded. There are two independent authors (Hjx and Zss). They performed literature searches, read titles and abstracts, and screened full texts independently, without communication with each other. If there were discrepancies or differences of opinion between the two authors, they discussed and resolved them through consensus. In cases where consensus could not be reached, a third author (Wss) was consulted to make the final decision. The flowchart of the search strategy is in Fig. 1.

### Data extraction

In our meta-analysis, two investigators (Hjx and Zss) collected and analyzed a range of data from the literature independently, including the first author’s name, publication year, patient age, sample size, study type, type of operation, timing, and duration of medication, treatment plan, method of delirium evaluation, the incidence of delirium, operation time, extubation time, and incidence of hypotension and low heart rate. By examining this wide range of variables, we aimed to conduct a comprehensive evaluation of the effects of different anesthetic drugs on postoperative delirium.

### Definition of outcomes

We considered the incidence of postoperative cognitive dysfunction (POCD) as the primary index, and the incidence of hypotension, sinus bradycardia, operation time, and extubation time as secondary indicators. Hypotension was defined as a systolic blood pressure lower than 90 mmHg or diastolic blood pressure lower than 60 mmHg, while sinus bradycardia was defined as a heart rate less than 60 beats per minute.

### Assessment of risk of bias

Randomized controlled trials (RCTs), considered the gold standard for verifying the effectiveness of interventions, are an integral part of producing high-quality meta-analyses. To further ensure the quality of the studies included in our meta-analysis, we utilized the Cochrane Risk of Bias (ROB) tool-2 to evaluate the risk of bias in each study. Five domains for assessing the risk of bias: random sequence generation, allocation concealment, blinding, incomplete outcome data, and selective reporting. Bias risk assessment: high, medium, or low risk of bias is assigned for each assessment item, and bias risk messages are generated accordingly. This evaluation was conducted independently by two authors (Hjx and Zss), to reduce the potential for bias in the assessment process.

### Statistic analysis

For continuous variables in the outcome index, we expressed them as mean ± standard deviation. However, if the variable is expressed as median (interquartile range), and the sample size is large enough and close to the normal distribution, we can treat the median as the mean, and calculate the standard deviation by taking the quartile range and dividing it by 1.35 For dichotomous variables, we extracted the number of occurrences and the total number of the sample.

We used I^2^ and *p*-value to estimate the statistical difference, where I^2^ > 50%, and *p* < 0.05, were considered significant heterogeneity using STATA (version 15). We created interval diagrams for all comparative and predictive confidence intervals and objectively assessed each variable using a network graph. To evaluate potential discrepancies between direct and indirect comparisons, we compared loop inconsistencies in our analysis. we also used the node splitting method and consistency model to test for consistency.

We sorted the intervention measures, and the area under the curve indicates the level of the intervention. The effect of treatment of interventions is ranked according to the area under the curve ranking SUCRA. To evaluate publication bias, we used a funnel plot.

## Results

### Study selection

As shown in Fig. 1, we initially identified a total of 4012 articles from three databases (PubMed, Cochrane, and EMBASE). After excluding 442 duplicates, 2059 irrelevant studies, 577 studies on other topics, 234 animal experiments, 10 non-English studies, and 604 abstracts, letters, or conference proceedings, we proceeded with the full-text screening of the remaining 86 articles.

Out of the 86 articles, we excluded 7 studies that did not involve general anesthesia, 11 that were not RCTs, 15 that did not report the incidence of delirium, 4 that had incomplete data, and 8 that were limited to a specific age group. Finally, out of the 41 studies that met the inclusion criteria, we conducted our meta-analysis.

### Study characteristics

**Fig. 2:**
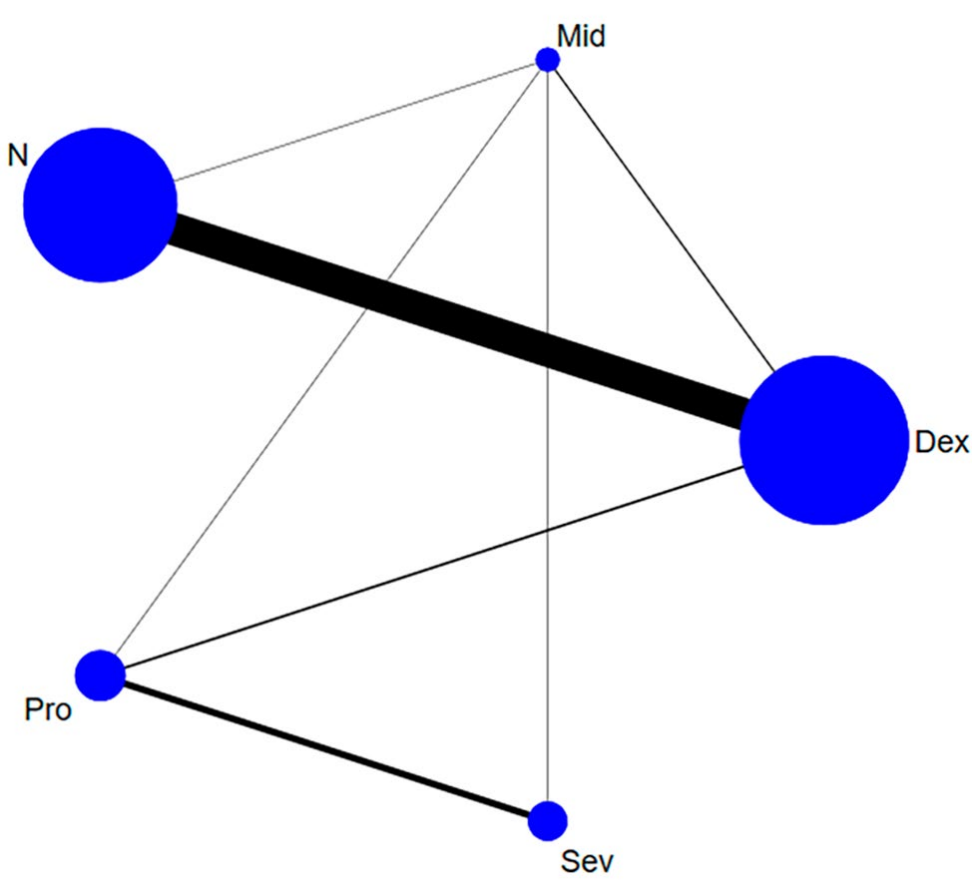
network plot: The relationship between delirium incidence and the overall structure of a network can be described as network geometry

As presented in (PS: Table 1), the studies included in our meta-analysis were published between 2014 and 2022. Among the 41 studies, 12 were focused on cardiac surgery, 28 were focused on non-cardiac surgery, and 1 study included both cardiac and non-cardiac surgery. Furthermore, 6 studies were conducted in the ICU, 33 studies were conducted in the OR, and 2 studies included both OR and ICU settings. Figure 2 displays the network plot.

### Risk of bias within studies

**Fig. 3:**
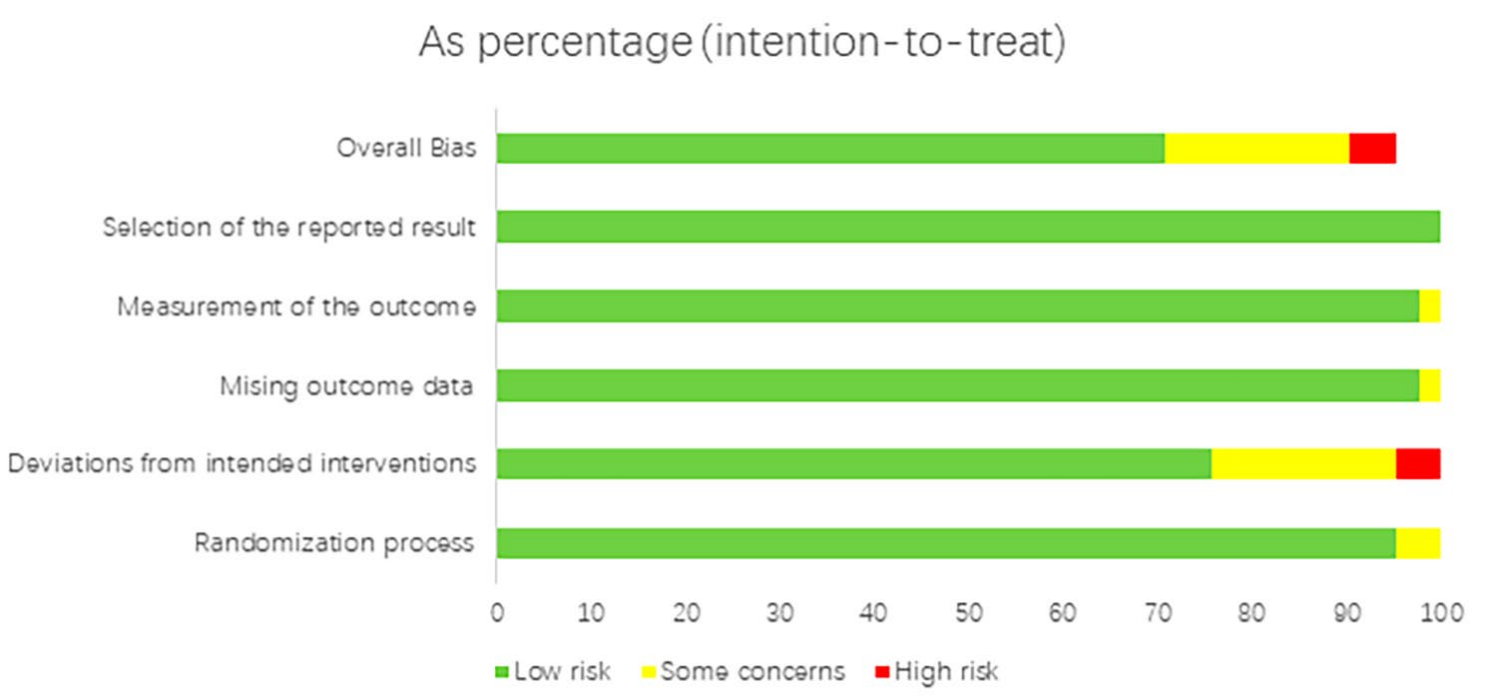
Literature quality evaluation Document quality is evaluated using ROB-2

In our assessment, we identified two studies as high-risk [20, 21], and we determined that two studies posed a potentially high risk of bias. One study lacked sufficient information regarding the implementation of a double-blind procedure. The other study reported that anesthesiologists and nurses were not blinded due to the need to adjust the timing and dosage of dexmedetomidine. In addition, we considered five studies to have a medium risk of bias [22–26]. The researchers and subjects may not be double-blind and There is no mention of whether there was an appropriate analysis used to estimate the effect of assignment to intervention (Fig. 3).

## Synthesis of results

### Incidence of postoperative delirium and postoperative cognitive dysfunction

**Fig. 4:**
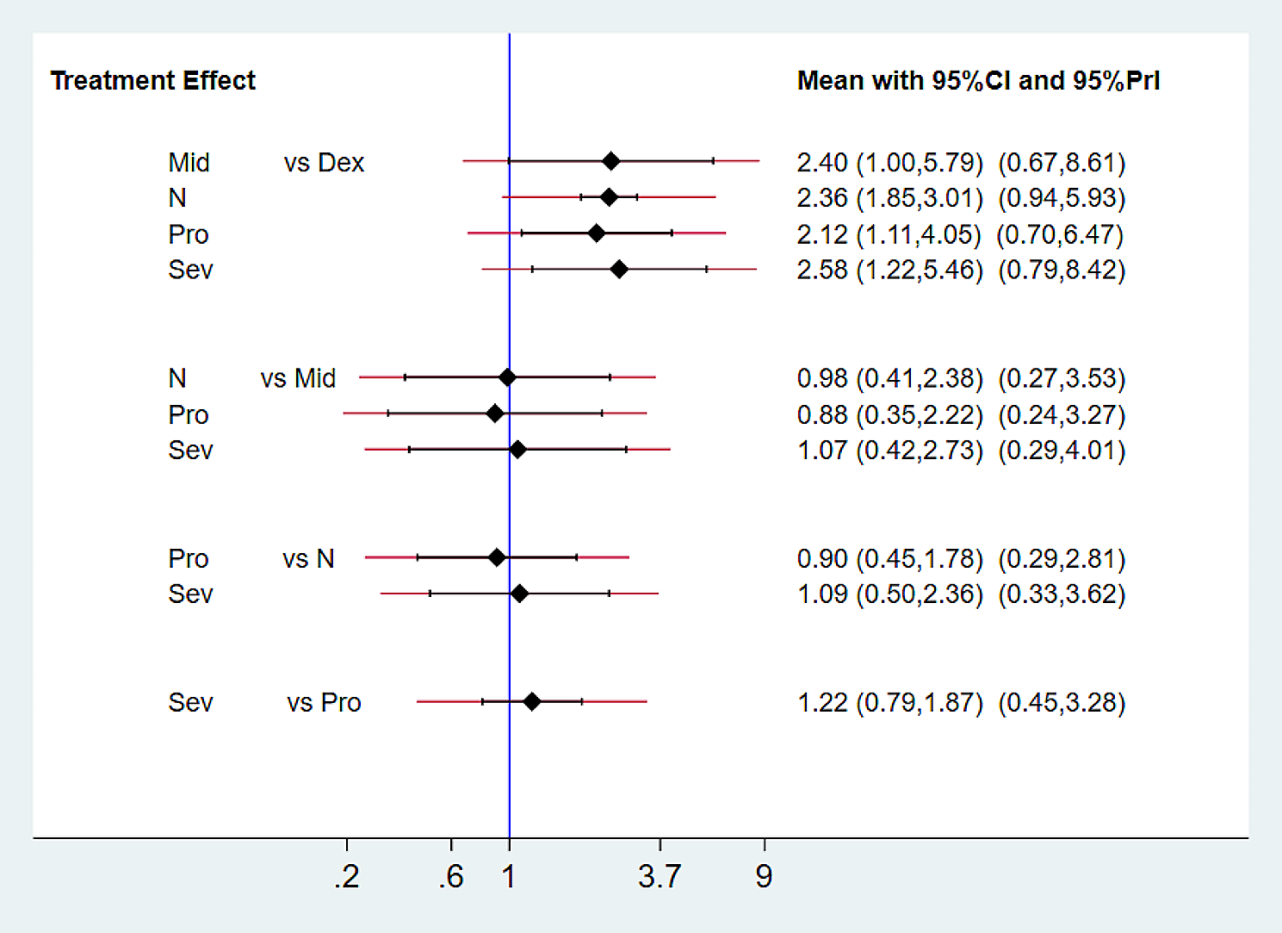
The pairwise comparison interval diagram. the comparison of several interventions in terms of their effectiveness in reducing postoperative delirium and Postoperative cognitive dysfunction

The pairwise comparison interval diagram (Fig. 4) presents the comparison of several interventions in terms of their effectiveness in reducing postoperative delirium and postoperative cognitive dysfunction. The diagram shows that midazolam (OR 2.40; 95% CI 1.00 to 5.79), propofol (OR 2.12; 95% CI 1.11 to 4.05), sevoflurane (OR 2.58; 95% CI 1.22 to 5.46), and saline(OR 2.36 95%CI 1.85–3.01) are less effective in reducing postoperative delirium and postoperative cognitive dysfunction when compared to dexmedetomidine. A league chart (PS Fig. 1) is also provided for further information. We have summarized a forest plot comparing multiple drugs (PS Fig. 2).

### Extubation time

Several factors have been identified as potential risk factors for postoperative delirium, and the duration of extubation is one such factor. propofol was found to have a higher likelihood of causing postoperative delirium and prolonged extubation time when compared to dexmedetomidine (MD 350.58 95% CI 323.78-377.38), midazolam (MD 344.28 95%CI 316.98-371.57), and normal saline (MD 351.21 95% CI 324.32–378.10). sevoflurane with a higher incidence of postoperative delirium with longer extubation times when compared to dexmedetomidine (MD 349.58 95% CI 322.27-376.88), midazolam (MD 343.28 95% CI 315.49-371.07), physiological saline (MD 350.21 95% CI 322.82–377.60).

### Operation time

A total of 30 articles were included in the study, comprising 4924 participants. Operation time is also one of the causes of delirium, but affected by many other factors, there is no significant difference in the effect of different operation times and drugs on postoperative delirium.

## Results of subgroup analyses

### Operation time

To identify subgroups of patients who may benefit more from measures aimed at preventing postoperative delirium, the study participants were divided into two groups based on whether their surgery lasted longer or shorter than three hours.

#### Operation time < 3 h

The incidence of postoperative delirium was significantly lower among patients who underwent surgery lasting less than three hours when received dexmedetomidine (OR 2.74 95% 1.48–5.05) compared to those who received normal saline as part of the anesthesia regimen.

#### Operation time > 3 h

When surgical procedures lasted longer than three hours, it was found that the preventative effect of administering dexmedetomidine to reduce the incidence of postoperative delirium was significantly better than that of using another part of the anesthesia regimen (PS Fig. 3).

### Extubation time

We categorized extubation time into less than 1 h and more than 5 h.

#### Extubation time < 1 h

It was observed that administering dexmedetomidine within the first hour after surgery was better than the usage of midazolam (OR 15.55 95%CI 1.11-218.13) and normal saline (OR 3.48 95%CI 1.75–6.94) preventing postoperative delirium.

#### Extubation time > 5 h

when extubation time exceeded 5 h, the use of propofol (OR 2.20 95% 1.23–3.93) and normal saline (OR 2.63 95%CI 1.09–6.31) was found to be less effective compared to the usage of dexmedetomidine in preventing postoperative delirium.

### Type of surgery

During our study, we divided participants into cardiac and non-cardiac groups.

#### Cardiac surgery

Among the cardiac group, a total of 12 articles were included in the study and 4 drugs were compared (propofol, sevoflurane, dexmedetomidine, saline). it was observed that patients who received midazolam (OR 3.34 95%CI 2.04–5.48) and normal saline (OR 2.27 95%CI 1.17–4.39) were more likely to develop postoperative delirium compared to those who received dexmedetomidine.

#### Non-cardiac surgery

A total of 28 articles were included in the study, which compared 5 drugs. Our findings suggest that the use of normal saline in cardiac surgery is more likely to contribute to postoperative delirium compared to the usage of dexmedetomidine (OR 1.98 95%CI 1.44–2.71).

### Results of secondary indicators

Bradycardia was reported in 12 articles that included a total of 1831 participants. The incidence rate of bradycardia was significantly lower in the saline group compared to the dexmedetomidine group (OR 0.55, 95% CI 0.37 to 0.80). However, no significant difference was observed in the incidence of heart rate among other sedatives.

Out of the 41 articles analyzed, only 12 contained records of hypotension. The rates of hypotension for several drugs were not found to be statistically significant.

### SUCRA probability ranking

**Fig. 5:**
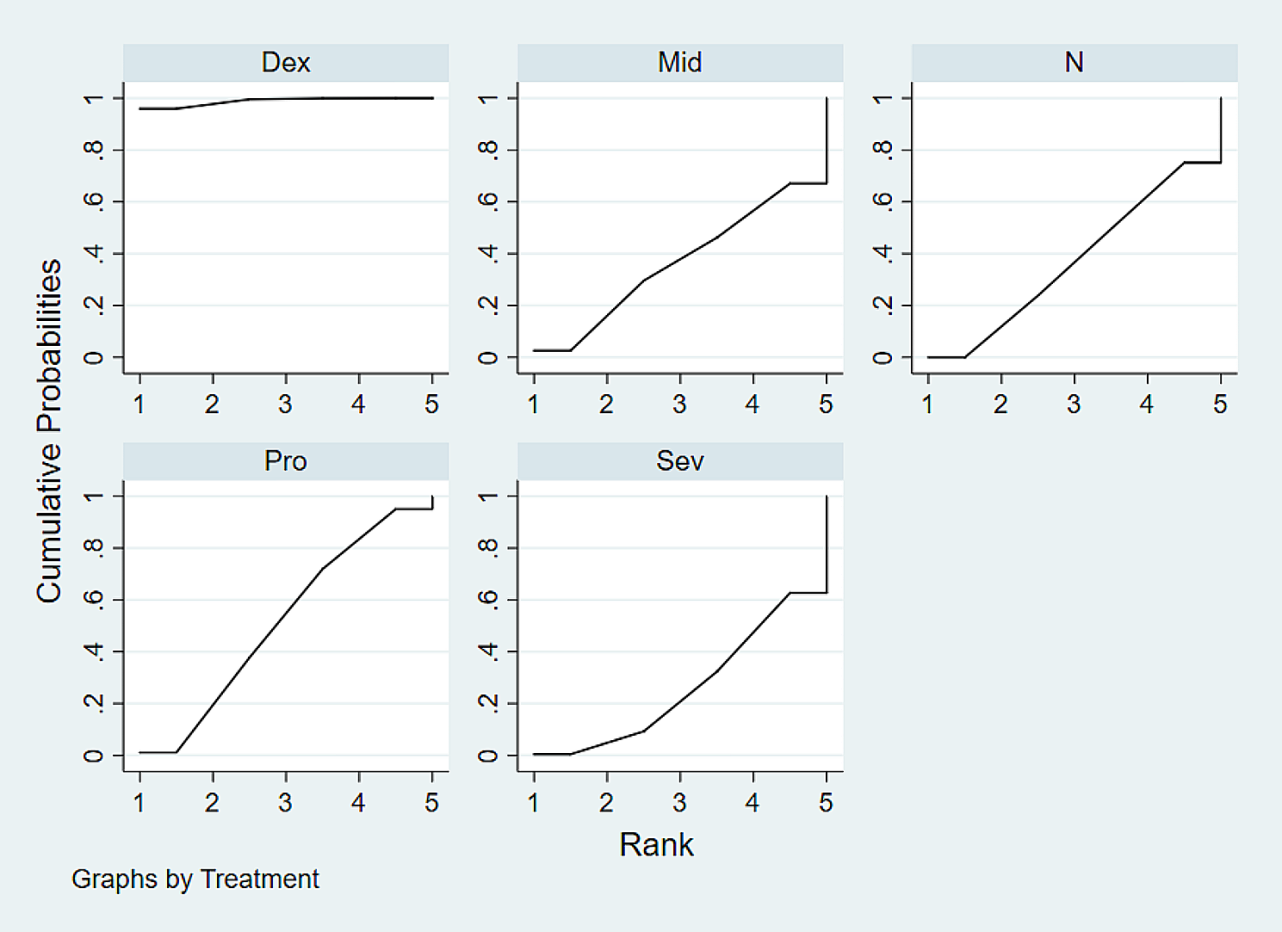
The Ranking Probabilities Diagram. The product map under the curve is also shown in the figure. A larger area under the curve indicates a greater possibility of the best pre-measures. Abbreviations used in the chart include Dex for Dexmedetomidine, Mid for Midazolam, N for Saline, Pro for Propofol, and Sev for Sevoflurane

According to the Ranking Probabilities Diagram (Fig. 5), dexmedetomidine was ranked the highest among all the interventions, indicating that it may be the most effective among the drugs studied. The SUCRA indices were found dexmedetomidine (98.8%) > Propofol (51.4%) > saline (37.2%) > Midazolam (36.4%) > Sevoflurane (26.2%), indicating that dexmedetomidine has the highest probability of being the most effective intervention for reducing the incidence of postoperative delirium.

### Publication bias

**Fig. 6:**
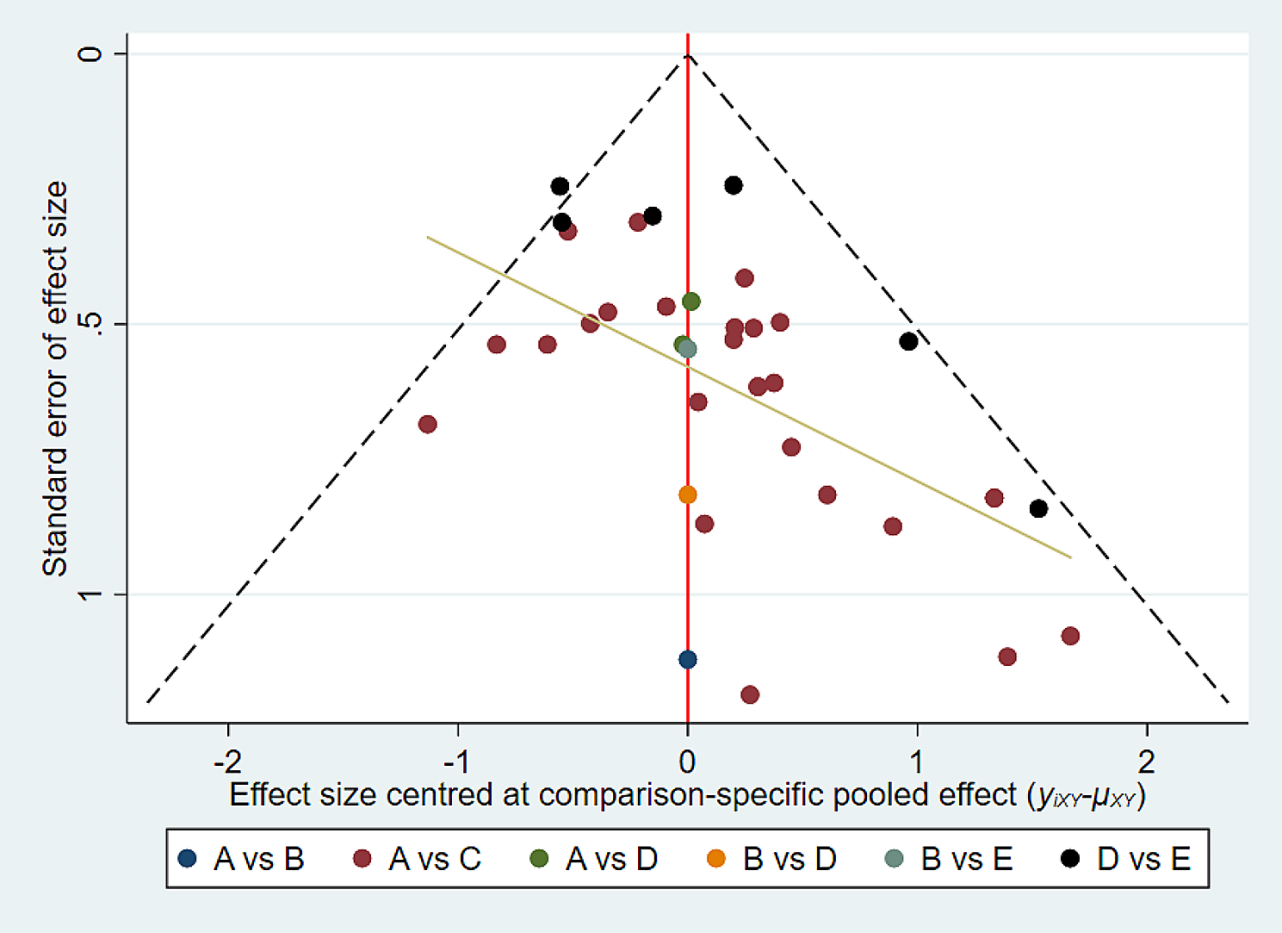
The funnel plot. a funnel chart shows that the distribution of each study is roughly symmetrical the effect OR is plotted on the abscissa, while the reciprocal 1/SE (log OR) of the standard error of the effect is plotted on the ordinate. The dotted lines perpendicular to the horizontal axis represent the combined effect, and the dotted lines on both sides of the chart represent the 95% confidence interval Acronym: (Dex): dexmedetomidine; (Mid): midazolam; (N): saline; (Pro): propofol; (Sev): sevoflurane; A:(Dex):dexmedetomidine; B:(Mid):midazolam; C:(N):saline; D:P(Pro):propofol; E(Sev):sevoflurane;

We used funnel plots to compare the differences in the mean changes of all outcome measures between the treatment group and the placebo group. The majority of the scatter points in all funnel plots were located on both sides of the vertical line. The funnel plot (Fig. 6) shows that the distribution of each study is roughly symmetrical, which suggests the absence of publication bias or other forms of bias.

### Comparison of consistency and heterogeneity

The following Stata code “network meta I (*P* = 0.18 *P* > 0.05)” indicates that a consistency model can be applied for the analysis. Additionally, the results of the node splitting map (PS Fig. 4) indicate that there is no apparent local inconsistency for both direct and indirect comparisons(all *P* > 0.05), demonstrating the absence of significant heterogeneity. In The ring inconsistency (PS Fig. 5), midazolam, propofol, and sevoflurane were compared with each other regarding their ability to undergo cyclization (*P* > 0.05), dexmedetomidine, midazolam, and propofol have the ability to form a ring structure through cyclization (*P* > 0.05), test also verifies that there is no obvious inconsistency between direct comparison and indirect comparison.

## Discussion

The incidence of postoperative delirium with dexmedetomidine was found to be lower than that of a placebo, propofol, sevoflurane, and midazolam, regardless of whether the extubation time was longer than 1 or 5 h, the operation time was less than 3 h or more than 3 h, or whether the surgery was cardiac or non-cardiac. This indicates that dexmedetomidine has a positive effect on preventing postoperative delirium. However, it should be noted that dexmedetomidine carries a higher risk of reducing heart rate than normal saline. Therefore, it is necessary to closely monitor heart rate when using dexmedetomidine and discontinue the drug promptly if necessary.

Our analysis supports previous research and guidelines [27], indicating that dexmedetomidine can play a positive role in preventing postoperative delirium [28, 29]. A large randomized study showed that dexmedetomidine does not reduce the incidence of postoperative delirium. It is possible that other side effects, such as hypotension, could contribute to the occurrence of postoperative delirium. Therefore, the conclusion can be drawn that there is no significant difference in the rate of postoperative delirium between the dexmedetomidine group and the normal group. Furthermore, it is important to acknowledge that postoperative delirium can be caused by multiple factors, which necessitates a comprehensive and dialectic evaluation. dexmedetomidine functions by inhibiting the release of norepinephrine through the activation of α2 receptors in the brain, which reduces the excitability of neurons and enhances the inhibitory effect of γ-aminobutyric acid (GABA). This mechanism can provide analgesic effects, relax patients, and reduce anxiety [30]. Dexmedetomidine can also improve postoperative sedation and sleep quality, and reduce sensory perception, nerve exhaustion, and stress response, promoting better patient recovery [31]. Finally, dexmedetomidine may have a positive effect on the prevention of postoperative delirium by regulating the inflammatory response and metabolic activity of neurons in the brain, thus exerting anti-inflammatory and neuroprotective effects [20, 32]. Therefore, the use of dexmedetomidine is effective in preventing postoperative delirium.

Our analysis found that compared to saline, midazolam, sevoflurane, and propofol, dexmedetomidine is more likely to cause bradycardia, but there was no significant difference in the incidence of hypotension among all sedatives. We identified 12 articles reporting bradycardia and 12 articles reporting hypotension among the total of 41 articles included in our analysis. However, due to the limited number of articles available on hypotension and bradycardia, we do not have sufficient evidence to establish the differences between different sedatives. Clinicians should consider the potential side effects when administering drugs to patients.

According to some guidelines, the duration of surgical procedures is recognized as one of the factors that affect the occurrence of postoperative delirium. However, different studies appear to have arrived at varying conclusions regarding the specific length of time required to increase the risk of developing this condition [33]. Some studies have suggested that the rate of postoperative delirium increases significantly if the surgical procedure takes more than 2 h [34], while others suggest that the threshold could be 3, 4, or even 5 h [35–37]. In our study, we observed that when the duration of surgery exceeded three hours, administering dexmedetomidine had a significantly greater effect in preventing postoperative delirium compared to the other anesthesia regimen. The incidence of postoperative delirium was significantly reduced in surgeries lasting less than three hours, where patients were administered dexmedetomidine compared to the usage of normal saline as part of the anesthesia regimen. The specific duration and threshold for postoperative delirium risk may depend on factors such as the type and location of surgery, the patient’s health status, and anesthesia-related factors. Therefore, clinicians need to consider various risk factors carefully for each patient and surgical procedure to minimize the risk of postoperative delirium.

Extubation is the process of removing a breathing tube that is inserted into a patient’s airway during surgery to help them breathe. The longer the duration of extubation (the amount of time the breathing tube is in place), the higher the risk of postoperative delirium [38, 39]. This is because the tube can cause irritation and inflammation in the airways, which can trigger an inflammatory response in the body that can lead to cognitive issues [40, 41]. A study looked at the relationship between extubation duration and postoperative delirium in elderly patients undergoing cardiopulmonary bypass. The study found that patients who had a longer duration of extubation were more likely to develop postoperative delirium, compared to patients who had a shorter duration of extubation. Our study found that the extubation time for patients who received propofol and sevoflurane was longer compared to those who received dexmedetomidine, midazolam, and normal saline. Previous studies have demonstrated that the use of dexmedetomidine in intubated patients has a sedative effect, reducing restlessness and preventing postoperative delirium [42–45]. Our study further supports this finding, as we observed a positive effect of dexmedetomidine in preventing postoperative delirium regardless of the length of intubation.

Numerous studies have demonstrated that the use of dexmedetomidine can decrease the incidence of postoperative delirium [16, 46–48], although a few have reported otherwise [49, 50]. Postoperative delirium is associated with various factors such as the type of operation, age, the patient’s overall health status, excessive blood loss, and abnormal liver and kidney function. While dexmedetomidine has primarily been studied in the context of cardiac surgery, some research has shown that it can also be effective in preventing postoperative delirium in non-cardiac surgical procedures [51–54]. Our study provides further evidence in support of dexmedetomidine’s effectiveness, finding that it can reduce the incidence of postoperative delirium not only after cardiac surgery but also after non-cardiac surgical procedures.

This study has several strengths. First, this meta-analysis updates results from clinical studies over the past two years, thus several recently published, large-scale, and high-quality RCTs have been included. Second, to increase the credibility of the study, our study excluded clinical experiments with the number of participants in each group being less than 20. Finally, we also increased the effect of intubation time and operation time on postoperative delirium.

Our net-meta analysis has some limitations that need to be addressed. Firstly, some studies have smaller sample sizes, with only 40 subjects, which could affect the generalizability of the findings. Secondly, we only compared four sedatives, while there are likely more than four drugs used in clinical settings. Thirdly, postoperative delirium is multi-factorial and can be influenced by several variables such as age, type of operation, operation time, and intraoperative medication. Our analysis did not consider these factors. Lastly, the scale of postoperative delirium is subjective, which could influence the accuracy of the results.

## Conclusion

In conclusion, our findings suggest that dexmedetomidine has a positive effect in preventing postoperative delirium. However, the limitations of our analysis in terms of the literature included and the impact of dexmedetomidine on heart rate should be considered carefully when making clinical decisions. Besides, it is important for clinicians to carefully monitor the duration of extubation and operation and consider strategies to minimize the duration of intubation when possible, especially in older patients who are at higher risk for postoperative delirium. This may include optimizing pain control, managing anxiety and agitation, and providing supportive care to minimize irritation and inflammation in the airways. Moving forward, we hope to discover more drugs that can effectively prevent postoperative delirium.

### Electronic supplementary material

Below is the link to the electronic supplementary material.

**Supplementary Material 1:** PRISMA 2020 Checklist

**Supplementary Material 2: PS Fig 1** the league chart provided for further information about in reducing postoperative delirium and Postoperative cognitive dysfunction in 5 drugs

**Supplementary Material 3: PS Fig 2** the forest plot comparing multiple drugs

**Supplementary Material 4: PS Fig 3** operation more than 3h. Comparison of the incidence of postoperative delirium among five drugs when the operation time is more than 3 hours

**Supplementary Material 5: PS Fig 4** the ring inconsistency verifies that there is no obvious inconsistency between direct comparison and indirect comparison

**Supplementary Material 6: PS Fig 5** the node splitting map indicate that there is no apparent local inconsistency for both direct and indirect comparisons

**Supplementary Material 7: PS Table 1** the Study characteristics included in our meta-analysis were published between 2014 and 2022

## Data Availability

The datasets used and/or analysed during the current study available from the corresponding author on reasonable request.
