# Supplementary figures and images for: The role of perioperative sedative anesthetics in preventing postoperative delirium: a systematic review and network-meta analysis including 6679 patients
Source: BMC Cardiovasc Disord. 2024 Mar 6;24:147. doi: 10.1186/s12872-024-03783-5 (PMC10916082; doi:10.1186/s12872-024-03783-5)

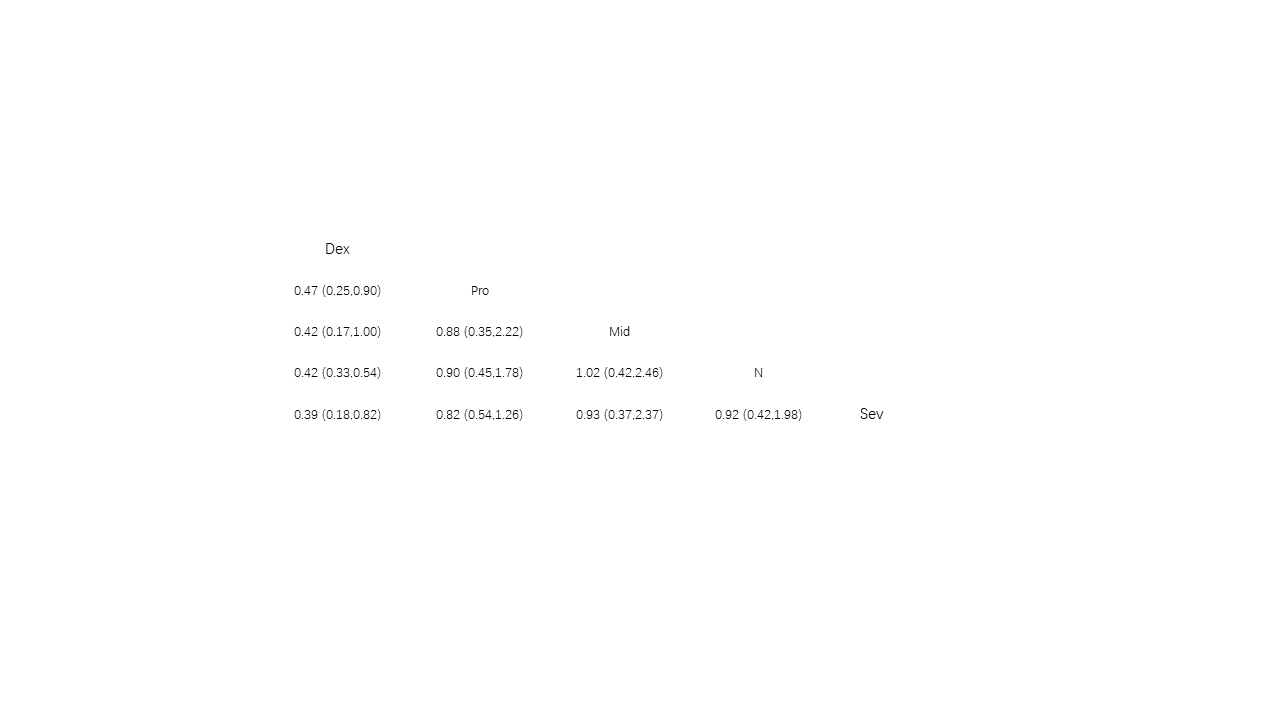

Supplement: Supplementary file 2 — Supplementary Material 2: PS Fig 1 the league chart provided for further information about in reducing postoperative delirium and Postoperative cognitive dysfunction in 5 drugs [file 12872_2024_3783_MOESM2_ESM.png]

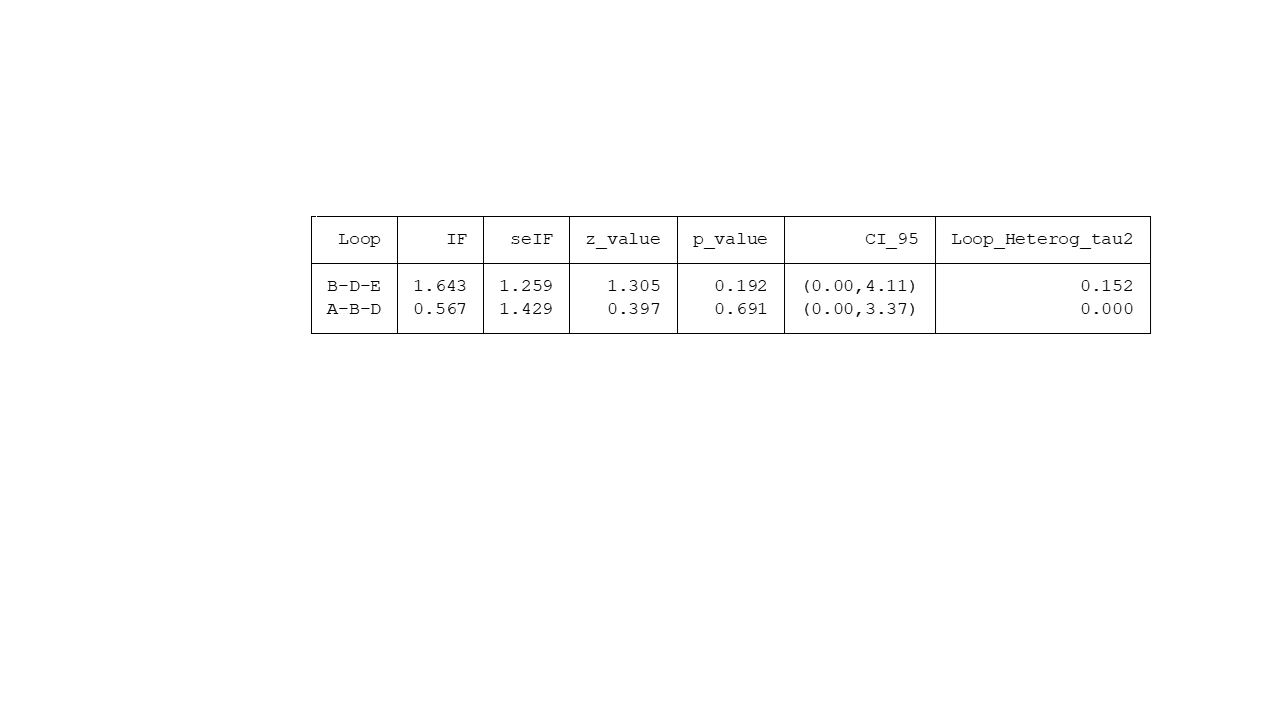

Supplement: Supplementary file 3 — Supplementary Material 3: PS Fig 2 the forest plot comparing multiple drugs [file 12872_2024_3783_MOESM3_ESM.png]

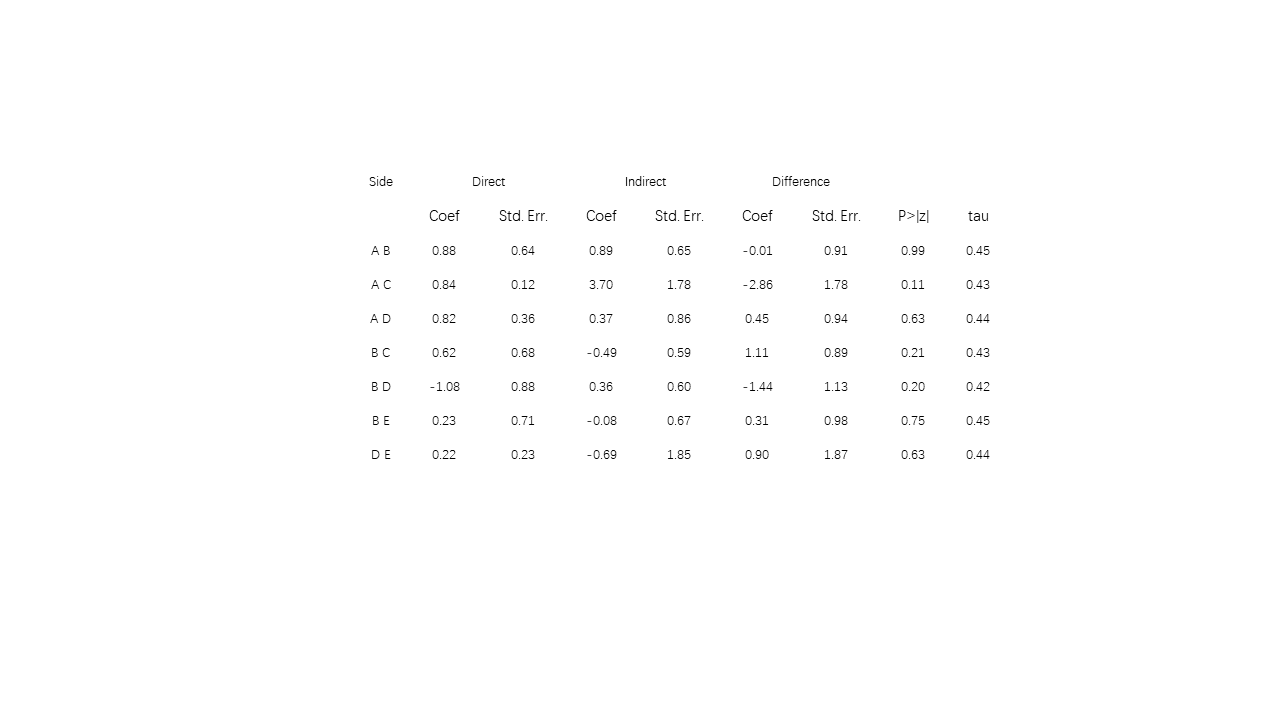

Supplement: Supplementary file 4 — Supplementary Material 4: PS Fig 3 operation more than 3h. Comparison of the incidence of postoperative delirium among five drugs when the operation time is more than 3 hours [file 12872_2024_3783_MOESM4_ESM.png]

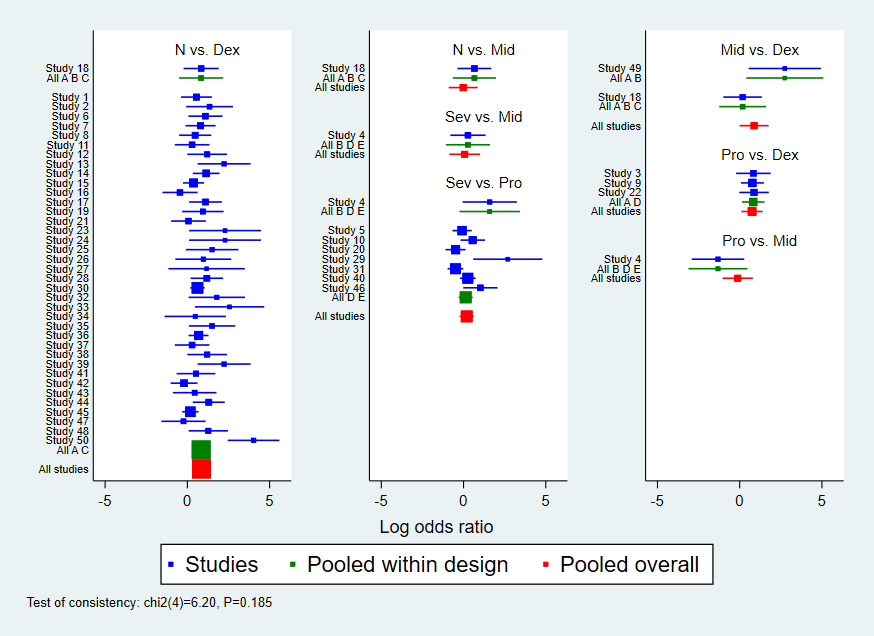

Supplement: Supplementary file 5 — Supplementary Material 5: PS Fig 4 the ring inconsistency verifies that there is no obvious inconsistency between direct comparison and indirect comparison [file 12872_2024_3783_MOESM5_ESM.png]

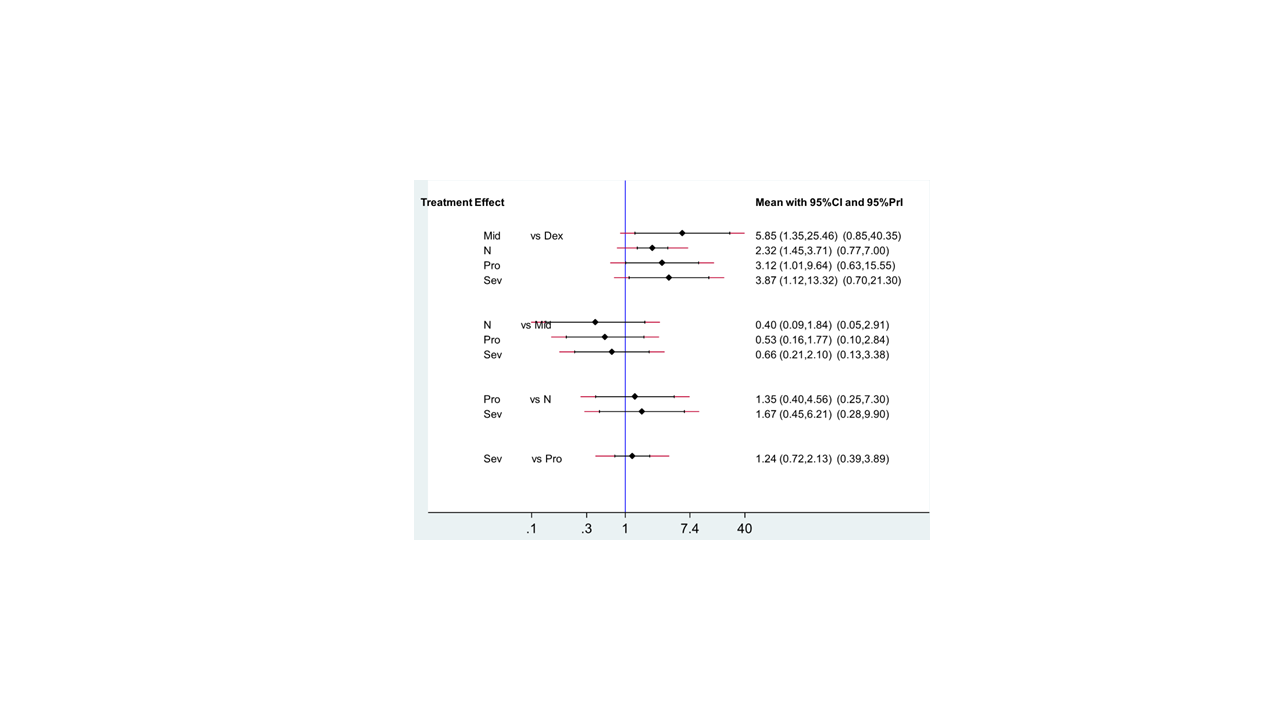

Supplement: Supplementary file 6 — Supplementary Material 6: PS Fig 5 the node splitting map indicate that there is no apparent local inconsistency for both direct and indirect comparisons [file 12872_2024_3783_MOESM6_ESM.png]
